# Spring Coughs

**Published:** 1908-04

**Authors:** James Burke

**Affiliations:** Mantowac, Wis.


### SPRING COUGHS.

BY JAMES BURKE, M. D., MANTOWAC, WIS.

It is irrational to treat a cough with opium or any of its derivatives, unless the several principles of which the opium is composed are isolated and available; then, it is possible one of these principles is antagonistic to (chemically affinitive for) one of the dominant disturbing toxins causing the cough; if so the neutralization of that disturber is at our scientific command.

The habits and environment of the coughing person will aid us in determining the dominant toxins actively producing the bronchial irritation. The cough is the result of the chemical attack of a toxin on the tissues of the lung, and especially on the structure of the nerve supply, in its metamorphosis of passing through the several stages of its cycle till it arrives by slow processes at the status of a benevolent excretory product.

This chemical worry of the lung cells catolyzes those cells, so that their secretory and excretory products are changed—pathologic—and by contact with neighboring cells, induce in them a retrograde product both of secretion and excretion, creating abnormal affinities, even extending into the volume of the general circulation.

Things in rapid progression leads to serious deterioration of the condition of the blood and fluids; early neutralization of the initial disturbing toxin would be "a stitch in time." Sajons has demonstrated that the pituitary body presides over and governs the balance in maintaining health in the natural order of metabolism, and in safeguarding the system in time of peril from autotoxins, bacterial infections and their toxins; the pathologic invader must be in solution in the blood in order that the special sense function of the anterior pituitary may detect its presence.

Through the posterior part of the pituitary body through special nerve connection, the adrenals thyroid and pancreas are given a rush command to increase their output; the adrenals to increase the adrenopidase, of which hemoglobin consists to ninety-four per cent. of its volume. Oridase is also the active principle of life, whose presence alone energizes the systemic cellular system; the thyroid furnishes the opsonins; the pancreas furnishes the trysin necessary for the energized leucocytes to encompass the bacteria and toxins, and convert them into living granulations in harmony with the needs of the tissues, in their momentary distress.

Early neutralization of the toxins by giving affinitive, cognate vegetable principles will obviate a formidable array of the immunizing forces; these are thereby preserved for the normal balance of approaching health.

In all forms of chronic disease the immunizing power of the system has been overworked; the leucocytes are starved and unable to revert and synthesize the bacteria, toxins and leucomains into pabulum useful for the transmutation into normal tissue—they are mere carriers of bacteria and toxins, without the ability to aid in normal function.

In this complex condition of chronic invalidism the immunizing power is in abeyance, and science must come to the rescue, before the integrity of the sick cells become destroyed.

Therapeutic interference must convert the abnormal amino-acids of the badly digested protein, and more virulent toxins, into benevolent excretory products by administering the indicated alkaloids and other available active principles.

The cough may have originated from one of the many sources of production of auto-toxins. Start at the beginning, correct bad metabolic habits by relieving the functional cellular defect; re-establish normal alkalinity of the blood, by the proper use of calcium salts; interdict the ingestion of carbo-hydrates till the pancreas recuperates sufficiently to properly digest them, and to furnish trypsin to normally revert proteid food, and to enable the leucocytes to digest bacteria and toxins.

